# Compression socks enhance sensory feedback to improve standing balance reactions and reflex control of walking

**DOI:** 10.1186/s13102-021-00284-2

**Published:** 2021-06-02

**Authors:** Yao Sun, Bridget Munro, E. Paul Zehr

**Affiliations:** 1 Rehabilitation Neuroscience Laboratory, University of Victoria, PO Box 3010 STN CSC, Victoria, BC V8W 3P1 Canada; 2 Human Discovery Science, International Collaboration on Repair Discovery (ICORD), Vancouver, BC Canada; 3 Centre for Biomedical Research, University of Victoria, Victoria, BC Canada; 4 Nike Sport Research Laboratory, NIKE Inc., Beaverton, OR USA; 5 Division of Medical Science, University of Victoria, Victoria, BC Canada; 6 Zanshin Consulting Inc., Victoria, BC Canada

**Keywords:** Compression socks, Sensory modulation, Cutaneous reflexes, Walking, Balance control

## Abstract

**Background:**

Compression garments are generally used for their potential benefits in exercise performance and post-exercise recovery. Previous studies show that compression sleeves worn at the elbow change neuromuscular control and improve performance during reaching movement. Cutaneous stimulation of the foot skin produces location-specific reflexes in the lower limb that guide foot placement during locomotion. However, it is not clear whether enhancement of sensory feedback with compression socks can alter the neuromuscular excitability of muscles in the leg and amplify balance performance and walking. The current project aimed to determine whether enhanced sensory input from wearing compression socks could affect: 1) spinal cord excitability (as measured by cutaneous reflexes from stimulation at the top or bottom of the foot during locomotion); 2) static balance performance; and, 3) dynamic balance performance following virtual perturbations.

**Methods:**

Twelve participants completed walking and balance tasks wearing four types of garments: 1) non-compression (control) socks; 2) ankle compression socks; 3) calf-compression socks; and, 4) customized ankle sleeves. During walking, electrical stimulations were delivered to three discrete locations on the dorsal (ankle crease, forefoot medial) and plantar (forefoot medial) surfaces of the foot in separate trials with each garment. Electromyography of ankle dorsiflexor tibialis anterior, plantarflexor medial gastrocnemius and evertor peroneus longus were measured bilaterally along with kinematic data from knee and ankle and kinetics under the right (stimulated) foot.

**Results:**

Compared to control socks, altered cutaneous reflexes and biomechanical responses were observed in all the conditions during walking. In dynamic balance tests, time and integrated EMG for recovering from virtual perturbation were significantly reduced when wearing calf compression socks and the ankle sleeve.

**Conclusions:**

Our findings suggest sensory enhancement from compression garments modifies spinal cord excitability during walking and improves performance in balance recovery after virtual perturbation.

## Background

Sensory feedback from receptors in the skin and muscles play important roles in regulating movement. Compression garments, such as socks or leggings, are used in different activities for putative performance benefits, like increased anaerobic threshold during running [1], greater power output in jump tests after fatigue [2], and improved post-exercise recovery [3–6]. The mechanisms of these effects and relationships to sensory feedback are not currently well understood.

One of the major physiological changes caused by compression garments is altered sensory feedback. The potential effects of altered sensory feedback from compression garments on motor performance have been proposed in several studies. Kraemer and colleagues [2, 7] found improved power output in repetitive vertical jumps when participants wearing compression shorts. The authors suggested the enhancement is likely due to reduced muscle oscillation and enhanced joint awareness. Pearce et al. [8] compared performance in an elbow flexion/extension visuomotor tracking task and found significantly better performance in the group wearing an upper body full sleeve compression garment. Another study found that compression sleeves worn across the elbow improved accuracy of reaching and neural excitability at rest, during discrete reaching, and in a rhythmic arm cycling task [9]. Modulation of group Ia presynaptic inhibition is the presumed spinal mechanism for these effects. These results suggest that sensory input from compression apparel could affect movement accuracy and joint sensitivity at where compression is applied. It is presumed to result in an interaction between passively applied sensory enhancement and spinal cord reflex pathways assessed by traditional methods.

During locomotor activities, sensory feedback from cutaneous receptors plays a crucial role in modulating muscle activity to adapt to changes in the environment and prevent tripping and falling, such as “stumble corrective response”. Cutaneous reflexes in the leg have been extensively studied and show task- and phase-dependent reflex modulation [10–15]. Two earlier studies from the Zehr lab [12, 13] investigated the specific modulation of cutaneous reflexes by stimulating discrete regions in the dorsum and plantar side of the foot. Highly organized, topographic reflex effects were found in the lower limb muscles. Site and phase dependency were also found in kinematic and kinetic data. These findings suggest that sensory feedback from specific skin locations on the foot influence the general and specific mechanisms involved in regulating stance and swing phases of gait.

Use of compression garments to change spinal cord excitability could be measured as modulations of cutaneous reflexes and walking performance. While the effects of compression garments on cutaneous reflexes during locomotion have not been investigated, previous studies suggested that compression garments may affect postural control. Michael et al. [16] compared single-leg stance performance in female athletes wearing loose-fit and well-fitted compression stirrup leggings, and a control group wearing conventional shorts. Results showed that wearing well-fitted compression leggings significantly improved balance time and decreased postural sway during eyes-closed single-leg stance. Enhanced joint stability and proprioception obtained by using sports tape [17], which may be a proxy for compression apparel, are used extensively in sport or after injury. In a study by Karlsson and Andreasson [18], ankle taping significantly shortened the muscle response time in the peroneus longus to simulated ankle sprain on a tilting trapdoor.

Based on the current literature, this study aimed to explore the modulatory effect of compression socks on balance performance and spinal pathway excitability during walking. Two types of commercially available compression socks, one customized ankle sleeve, and one non-compression (“control”) socks were used. Measurements from the compression socks and ankle sleeve conditions were compared with data obtained while wearing control socks. We hypothesized that sensory feedback generated from the compression socks would alter the excitability of cutaneous pathways during locomotion and amplify the ability to respond to balance perturbation.

## Methods

### Participants

Twelve adults (8 females, 4 males; mean ± standard deviation age: 23 ± 3 years old, height: 173.1 ± 9.6 cm, weight: 72.6 ± 10.9 kg) without any neurological impairment or muscular injury in the past six months were recruited in a random sample.

### Experimental protocol

The study protocol was approved by the University of Victoria Human Research Ethics Board (protocol number: 16–138) and conducted in accordance with the Declaration of Helsinki. Consent forms were provided and signed by each participant before data collection.

To avoid fatigue, walking and balance tasks were performed on two separate days with randomized order. Both tasks were performed with wearing the same model of Nike shoes (Nike Free Trainer 3.0 V4) under one control condition with non-compression running socks (Nike Elite Cushioned, No-Show Tab) and three compression conditions using: 1) ankle compression socks (Nike Hyper Elite Cushioned), 2) calf compression socks (Nike Elite Graduated Compression, Over-the-Calf) and 3) customized ankle sleeve.

During walking tests, participants walked on a treadmill at a self-selected comfortable speed. Electrical stimulations were delivered to three discrete locations on 1) ankle crease, 2) plantar surface and 3) dorsal surface of the 1st metatarsal distal end of the right foot in three separate trials during each condition. During each trial, a total of 160 stimulations with 1–3 s interstimulus intervals were delivered to ensure stimulations are pseudorandomly distributed across the step cycle. Each step cycle was divided into 12 phases beginning with the heel contact of the right feet and ending with the subsequent right heel contact at the swing to stance transition. Phase 1 to phase 7 are stance phase and phase 8 to 12 are swing phase.

**Fig. 1:**
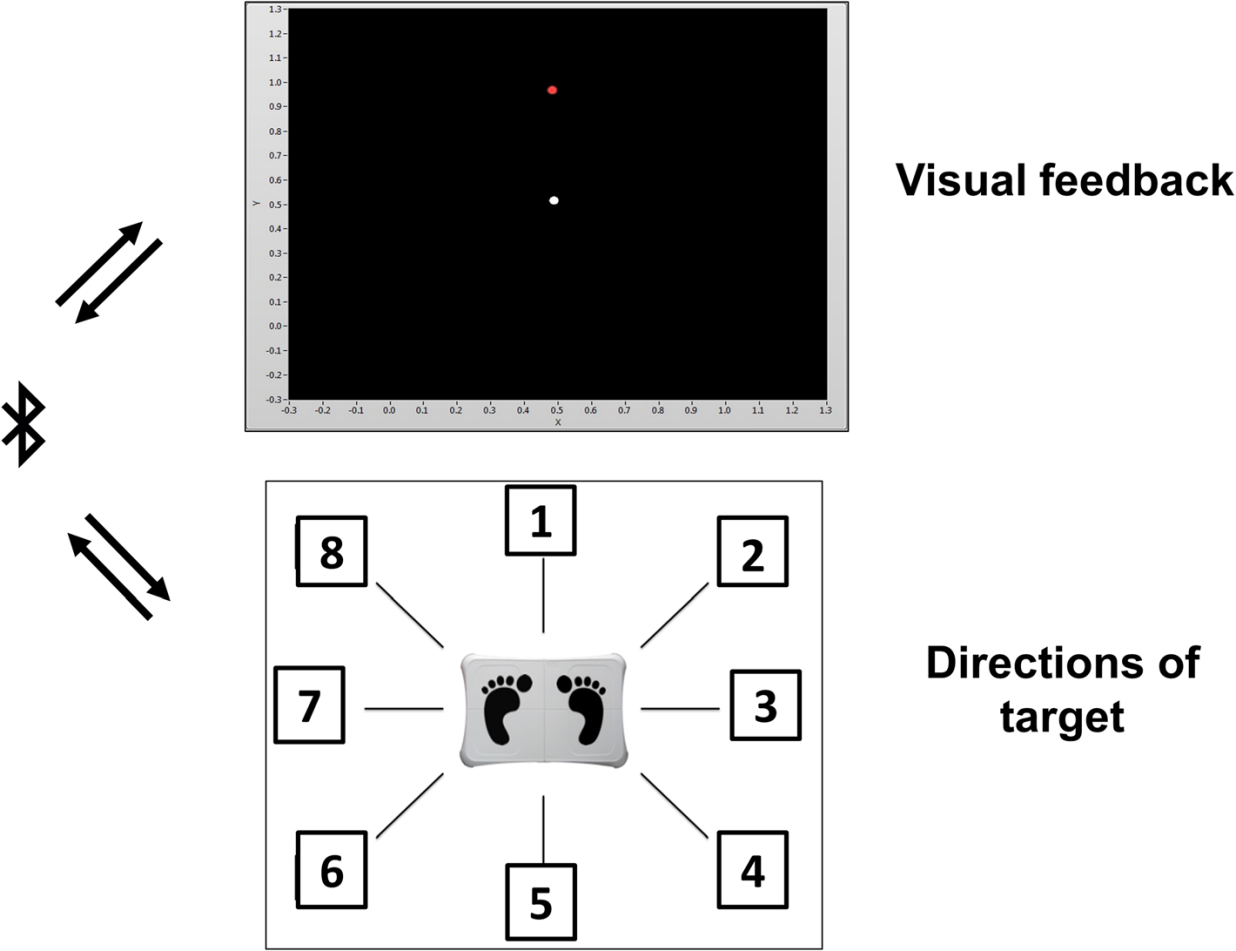
Dynamic balance test set-up. Top: Visual feedback on the computer screen. The white dot represents the participant’s center of pressure. The red dot represents the target. Bottom: Numbers around the force plate represent the eight directions of the target during dynamic balance test. Data from the force sensors were transmitted to a customized LabVIEW program through Bluetooth

Balance tests were performed by participants standing on a commercially available balance board (Nintendo Wii Balance Board). Signals from the four force sensors in the balance board were collected at a sampling rate of 100 Hz and the location of the center of pressure (COP) was calculated and recorded through a customized LabVIEW program. Static balance tests included double leg stance, single leg stance (with the non-dominant foot), and tandem stance (with the dominant foot at the front) from the modified Balance Error Scoring System (mBESS) [19, 20]. Each task was performed for 20 s with eyes closed in all four conditions [19, 20]. Dynamic balance performance was assessed by a customized LabVIEW program used in a previous study [21]. The customized program generated participants' COP dot and a target dot on the screen while participants standing on the balance board. Participants were instructed to move their COP dot to reach the target as quickly as possible by distributing their bodyweight. A detailed description of the dynamic balance test procedure can be found in the study from Sun & Cullen (2020) [21]. In the current study, one practice trial was performed at the beginning of the data collection and five trials were recorded under each condition. A schematic diagram of the dynamic balance test setup is illustrated in Fig. 1.

To confirm any potential effect of compression garments was not diminished by wearing shoes, an additional control trial was performed during balance tests with participants wearing control socks without shoes.

Pressures generated by each compression garment were measured on a Swisslastic leg by using a medical stocking pressure tester (MST Professional 2, Swisslastic AG, Switzerland). The circumference of the Swisslastic leg model was determined by the averaged leg circumferences of all the participants. Pressures were measured around 4 locations including ankle, midway between ankle to the maximum circumference of the calf, maximum circumference of the calf, and the top of the calf before the knee was measured. Pressures from each compression socks are presented in Table 1.

**Table 1:**
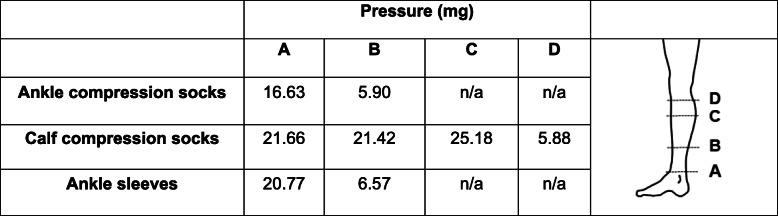
Pressures generated by compression socks. Pressures were measured at four different locations (A-D) as indicated in the diagram on the right

### Electromyography (EMG)

Muscle activity of tibialis anterior (TA), peroneus longus (PL), and medial gastrocnemius (MG) muscles were measured bilaterally during both walking and balance tests. After cleaning and preparing the skin, disposable surface electrodes (Thought Technology Ltd., Quebec, Canada) were placed on the test muscles. EMG signals were amplified (× 5000), bandpass filtered from 100 to 300 Hz (GRASS P511, Astromed-Grass Inc.), and sampled at 2000 Hz on a customized LabVIEW program (National Instruments, Austin, TX).

### Electrical stimulation during walking tests

At the beginning of each garment condition, perceptual threshold (PT), the lowest stimulus intensity to evoke a detectable tactile sensation, was determined for each stimulation site. Electrical stimulation was applied during walking with stimulation intensity set at 3 × PT to evoke a strong but tolerable tactile cutaneous perception during walking [12, 13]. Each stimulation is a train of five 1.0 ms pulses at 300 Hz. A total of 160 stimulations were delivered with a 1–3 s interval in each trial.

### Biomechanical measurements during walking tests

Three force sensing resistors (FSR) were attached to the right insole to record the forces produced under participant’s heel, medial and lateral forefoot, similar to previous studies [12, 13, 22]. Angular positions of the knee (flexion/extension), and ankle (dorsi/plantarflexion) were measured with electrogoniometers (Biometrics Ltd., Gwent, UK). FSR and electrogoniometer signals were amplified (× 5000) and collected at 2000 Hz during walking.

### Data analysis

Offline data were analyzed using customized written MATLAB programs (Version R2011b, The Mathworks, Natick, MA, USA). FSR data from the right heel was used to determine the start and end of each gait cycle and each step cycle was divided into 12 phases beginning with heel contact and ending with the subsequent heel contact of the same foot studies. EMG data were rectified and low-pass filtered using 4th order Butterworth filter with cut-off frequency at 100Hz. Data from FSRs and electrogoniometers were low-pass filtered using 4th order Butterworth filter with cut-off frequency at 20 Hz. For all the EMG, kinematic and kinetic responses, stimulated data were subtracted from averaged unstimulated data to yield a reflex trace. Net cutaneous reflexes at each phase were calculated as the average cumulative reflex EMG 150 ms (ACRE150) after stimulation. This method has been used to determine the overall excitation or inhibition effects after stimulation [13, 23]. Net reflex amplitudes were then normalized to that muscle’s maximal activation during an undisturbed gait cycle in the same trial. Biomechanical responses were calculated within a 140–190 ms window post-stimulus. Responses in plantar force were normalized to the peak value of each FSR during the averaged undisturbed gait cycle. Responses in joint angles were normalized to the joint range of motion (ROM) of the undisturbed gait cycle in the same trial.

Static balance performance was evaluated by the area of 95% confident ellipse of COP from each trial. For dynamic balance tests, duration (recovery time) and integrated EMG of each muscle of reaching the target and returning to the center were calculated at each direction. Integrated EMG was normalized to each individual’s maximal value in the control socks without shoe condition.

### Statistical analysis

Planned comparisons were used to compare all the measurements in compression garment conditions to the control condition. Measurements during walking include net cutaneous reflexes, responses in plantar force and joint angle at each phase. Balance measurements include recovery time and integrated EMG during dynamic balance tests and the area of 95% confidence ellipse during static balance tests.

To confirm the effects of compression garment were not mitigated by wearing shoes, all of the balance test measurements in control socks with shoes condition were compared to the without shoe condition by using paired t-test.

All statistical analyses were completed using SPSS software (IBM SPSS Statistic, V23. Armonk, NY, USA), statistical significance was set at *p* ≤ 0.05.

## Results

### Balance tests

**Fig. 2:**
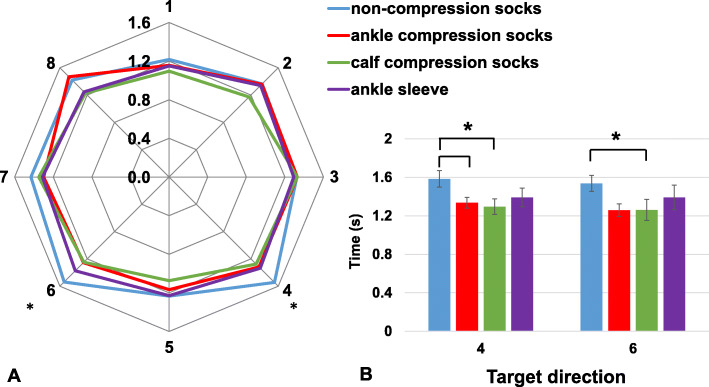
Group averaged recovery time following virtual perturbation during dynamic balance test. **a**: The numbers around the radar chart represent each target location. Radius represents the time (unit: seconds) used for moving the COP dot to the target and back to the center. **b**: Planned comparison results at target direction 4 and 6. Results from each condition are presented in different colours, blue: control socks; red: ankle compression socks; green: calf compression socks; purple: ankle sleeves. Error bars indicate standard error of the group. * represents significant difference between compression socks and control at *p* < 0.05

Compared to the control socks condition, calf compression socks reduced recovery time from 1.58 ± 0.39 s to 1.29 ± 0.28 s (*p* = 0.033, d = 0.856) and from 1.54 ± 0.27 s to 1.26 ± 0.27 s (*p* = 0.016, d = 1.012) when virtual perturbation was presented at direction 4 and 6 where participants had to lean to their posterior-rightward and posterior-leftward direction to change their COP. Ankle compression socks reduced recovery time from 1.58 ± 0.39 s to 1.33 ± 0.20 s (*p* = 0.016, d = 0.807) when the target was presented at direction 4. Recover time in dynamic balance assessments is presented in Fig. 2. Full results from each condition and statistical results are presented in Tables 2 and 3.

**Table 2:** Recovery time (mean ± standard deviation, unit: s) in dynamic balance test. Results show significant difference from control socks condition are in bold. Statistical results are presented in Table 3

	Target direction							
	1	2	3	4	5	6	7	8
**Control socks**	1.23 ± 0.30	1.37 ± 0.29	1.34 ± 0.33	1.58 ± 0.39	1.30 ± 0.35	1.54 ± 0.27	1.39 ± 0.37	1.40 ± 0.35
**Ankle compression socks**	1.18 ± 0.19	1.36 ± 0.23	1.32 ± 0.37	1.34 ± 0.20	1.18 ± 0.25	**1.26** ± **0.26**	1.30 ± 0.41	1.48 ± 0.39
**Calf compression socks**	1.11 ± 0.28	1.23 ± 0.38	1.33 ± 0.41	**1.29 ± 0.28**	1.10 ± 0.31	**1.26** ± **0.27**	1.36 ± 0.37	1.22 ± 0.32
**Ankle sleeves**	1.18 ± 0.34	1.36 ± 0.44	1.33 ± 0.31	1.39 ± 0.38	1.30 ± 0.38	1.39 ± 0.27	1.33 ± 0.41	1.29 ± 0.41

**Table 3:** Statistical outcomes of recovery time in dynamic balance test in compression garment conditions. *P*-values of planned comparison between control socks condition and each compression socks condition at eight directions of perturbation. * *p* ≤ 0.05

	Target directions							
	1	2	3	4	5	6	7	8
**Ankle compression socks vs. Control**	0.669	0.953	0.932	0.066	0.370	0.015*	0.555	0.614
**Calf compression socks vs. Control**	0.301	0.335	0.990	0.033*	0.157	0.016*	0.864	0.241
**Ankle sleeves vs. Control**	0.681	0.967	0.953	0.148	0.977	0.191	0.77	0.473

**Fig. 3:**
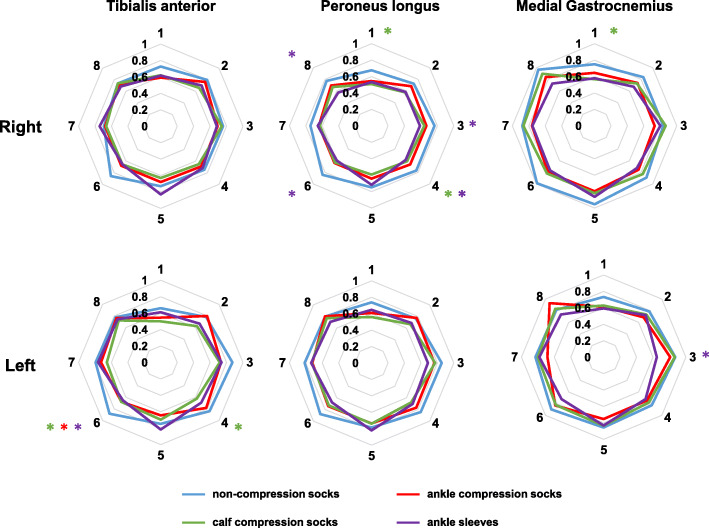
Group averaged integrated EMG following virtual perturbation during dynamic balance test. The numbers around each radar chart represent each target location. Radius represents the normalized integrated EMG when moving the COP dot to the target and back to the center. Results from each condition are presented in different colours, blue: control socks; red: ankle compression socks; green: calf compression socks; purple: ankle sleeves. * represents significant difference at *p* < 0.05. The colour of * represents the type of compression garment that significantly differed from control socks

Reduced integrated EMG was found in all compression garment conditions at different target directions (Fig. 3). With ankle compression socks, left TA integrated EMG was reduced (*p* = 0.048, d = 0.657) when the target was shown at direction 6. When wearing calf compression socks, reduced right PL (*p* = 0.042, d = 0.857) and right MG (*p* = 0.038; d = 0.863) integrated EMG were observed at target direction 1, where participants had to lean forward to adjust posture. Reduced right PL (*p* = 0.028, d = 0.921) and left TA (*p* = 0.032, d = 0.880) integrated EMG were observed at target direction 4. In ankle sleeve condition, reduced integrated EMG was mostly observed in the right PL muscle at target direction 3 (*p* = 0.035, d = 0.840), 4 (*p* = 0.013, d = 1.125), 6 (*p* = 0.029, d = 1.232), 8 (*p* = 0.011, d = 1.010). Ankle sleeve also reduced integrated EMG in the left MG (*p* = 0.026, d = 0.994) and left TA (*p* = 0.025; d = 1.535) muscle when the target was presented at direction 3 and direction 6 respectively. Results for all the muscles at each target direction are presented in Table 4. Planned comparison results are summarized in Table 5.

**Table 4:** Normalized integrated EMG (mean ± standard deviation) during dynamic balance test. Results show significant difference from control socks condition are in bold. Statistical results are presented in Table 5

		Target directions							
		1	2	3	4	5	6	7	8
**Control socks**	Right TA	0.73 ± 0.30	0.80 ± 0.28	0.76 ± 0.20	0.75 ± 0.28	0.73 ± 0.22	0.86 ± 0.45	0.69 ± 0.25	0.74 ± 0.24
	Right PL	0.68 ± 0.23	0.73 ± 0.23	0.77 ± 0.21	0.77 ± 0.20	0.75 ± 0.23	0.84 ± 0.41	0.74 ± 0.17	0.77 ± 0.17
	Right MG	0.75 ± 0.25	0.84 ± 0.30	0.84 ± 0.25	0.89 ± 0.22	0.95 ± 0.32	0.99 ± 0.54	0.88 ± 0.23	0.97 ± 0.22
	Left TA	0.66 ± 0.22	0.78 ± 0.29	0.87 ± 0.35	0.84 ± 0.32	0.75 ± 0.20	0.88 ± 0.38	0.79 ± 0.18	0.78 ± 0.23
	Left PL	0.74 ± 0.26	0.76 ± 0.20	0.85 ± 0.25	0.85 ± 0.23	0.79 ± 0.24	0.88 ± 0.36	0.81 ± 0.20	0.80 ± 0.22
	Left MG	0.73 ± 0.30	0.79 ± 0.30	0.87 ± 0.24	0.83 ± 0.22	0.86 ± 0.24	0.90 ± 0.36	0.83 ± 0.29	0.81 ± 0.26
**Ankle compression socks**	Right TA	0.59 ± 0.24	0.76 ± 0.25	0.69 ± 0.19	0.69 ± 0.16	0.68 ± 0.18	0.68 ± 0.18	0.67 ± 0.20	0.72 ± 0.19
	Right PL	0.54 ± 0.20	0.68 ± 0.18	0.67 ± 0.16	0.67 ± 0.16	0.64 ± 0.16	0.64 ± 0.19	0.64 ± 0.16	0.69 ± 0.18
	Right MG	0.65 ± 0.23	0.74 ± 0.20	0.73 ± 0.22	0.76 ± 0.27	0.80 ± 0.32	0.80 ± 0.19	0.76 ± 0.23	0.84 ± 0.32
	Left TA	0.54 ± 0.16	0.80 ± 0.21	0.73 ± 0.12	0.78 ± 0.16	0.65 ± 0.13	**0.68 ± 0.22**	0.73 ± 0.22	0.76 ± 0.10
	Left PL	0.61 ± 0.21	0.78 ± 0.25	0.76 ± 0.18	0.77 ± 0.22	0.74 ± 0.20	0.74 ± 0.26	0.72 ± 0.21	0.80 ± 0.20
	Left MG	0.61 ± 0.23	0.69 ± 0.20	0.81 ± 0.22	0.74 ± 0.23	0.75 ± 0.23	0.83 ± 0.30	0.68 ± 0.20	0.93 ± 0.38
**Calf compression socks**	Right TA	0.62 ± 0.26	0.66 ± 0.23	0.74 ± 0.26	0.65 ± 0.16	0.63 ± 0.14	0.66 ± 0.21	0.66 ± 0.21	0.74 ± 0.24
	Right PL	**0.51 ± 0.15**	0.58 ± 0.15	0.64 ± 0.20	**0.61 ± 0.14**	0.59 ± 0.14	0.63 ± 0.15	0.63 ± 0.18	0.67 ± 0.21
	Right MG	**0.57 ± 0.17**	0.73 ± 0.23	0.86 ± 0.32	0.83 ± 0.31	0.82 ± 0.34	0.82 ± 0.25	0.87 ± 0.27	0.90 ± 0.41
	Left TA	**0.50 ± 0.16**	0.62 ± 0.19	0.72 ± 0.23	**0.62 ± 0.13**	0.70 ± 0.19	**0.68 ± 0.16**	0.66 ± 0.17	0.72 ± 0.24
	Left PL	0.56 ± 0.26	0.67 ± 0.26	0.77 ± 0.36	0.68 ± 0.25	0.74 ± 0.29	0.73 ± 0.33	0.73 ± 0.30	0.77 ± 0.33
	Left MG	0.63 ± 0.23	0.74 ± 0.31	0.87 ± 0.28	0.78 ± 0.34	0.83 ± 0.33	0.82 ± 0.24	0.80 ± 0.23	0.83 ± 0.33
**Ankle sleeves**	Right TA	0.62 ± 0.24	0.70 ± 0.21	0.68 ± 0.37	0.72 ± 0.25	0.83 ± 0.27	0.66 ± 0.21	0.74 ± 0.31	0.69 ± 0.24
	Right PL	0.53 ± 0.20	0.58 ± 0.26	**0.59 ± 0.22**	**0.59 ± 0.18**	0.72 ± 0.32	**0.60 ± 0.24**	0.65 ± 0.32	**0.57 ± 0.19**
	Right MG	0.58 ± 0.17	0.67 ± 0.16	0.80 ± 0.27	0.73 ± 0.26	0.86 ± 0.30	0.77 ± 0.29	0.76 ± 0.25	0.73 ± 0.25
	Left TA	0.61 ± 0.22	0.67 ± 0.17	0.74 ± 0.24	0.69 ± 0.29	0.82 ± 0.24	**0.65 ± 0.14**	0.76 ± 0.29	0.74 ± 0.21
	Left PL	0.65 ± 0.27	0.69 ± 0.20	0.69 ± 0.24	0.71 ± 0.18	0.82 ± 0.24	0.68 ± 0.21	0.73 ± 0.26	0.71 ± 0.26
	Left MG	0.59 ± 0.25	0.72 ± 0.22	**0.65 ± 0.21**	0.72 ± 0.24	0.83 ± 0.26	0.72 ± 0.22	0.78 ± 0.18	0.73 ± 0.18

**Table 5:** Statistical outcomes of integrated EMG during dynamic balance test in compression garment conditions. *P*-values of planned comparison between control socks condition and each compression garment conditions at eight directions of perturbation. TA- tibialis anterior; PL-peroneal logus; MG- medial gastrocnemius. * p ≤ 0.05

		Target directions							
		1	2	3	4	5	6	7	8
**Ankle compression socks vs. Control**	Right TA	0.211	0.719	0.549	0.538	0.557	0.138	0.872	0.864
	Right PL	0.110	0.615	0.230	0.145	0.252	0.067	0.246	0.301
	Right MG	0.230	0.298	0.341	0.220	0.231	0.186	0.252	0.314
	Left TA	0.149	0.848	0.177	0.562	0.203	0.046*	0.465	0.846
	Left PL	0.219	0.897	0.420	0.392	0.635	0.262	0.355	0.983
	Left MG	0.257	0.357	0.525	0.441	0.354	0.569	0.166	0.345
**Calf compression socks vs. Control**	Right TA	0.323	0.173	0.896	0.290	0.251	0.089	0.780	0.997
	Right PL	0.042*	0.087	0.137	0.028*	0.096	0.057	0.201	0.175
	Right MG	0.038*	0.255	0.792	0.571	0.303	0.233	0.896	0.585
	Left TA	0.047*	0.089	0.137	0.032*	0.522	0.048*	0.134	0.501
	Left PL	0.090	0.302	0.466	0.070	0.654	0.225	0.390	0.770
	Left MG	0.327	0.636	0.946	0.640	0.848	0.522	0.781	0.886
**Ankle sleeves vs. Control**	Right TA	0.306	0.338	0.499	0.739	0.244	0.088	0.609	0.583
	Right PL	0.083	0.061	0.035*	0.013*	0.740	0.029*	0.314	0.011*
	Right MG	0.051	0.086	0.741	0.140	0.487	0.120	0.233	0.062
	Left TA	0.533	0.212	0.262	0.148	0.387	0.025*	0.740	0.672
	Left PL	0.386	0.415	0.131	0.124	0.723	0.101	0.425	0.366
	Left MG	0.190	0.557	0.026*	0.320	0.825	0.141	0.645	0.516

No significant effect of compression garment was found in static balance tests. The areas of 95% confidence ellipse in the compression garment conditions were not significantly different from the control socks condition during double leg stance, single leg stance and tandem stance tests. Planned comparisons results are summarized in Table 6.

**Table 6:** Statistical outcomes of the area of 95% confidence ellipse during static balance tests in compression garment conditions. *P-*values of planned comparison between control and each compression socks conditions

	Double-leg stance	Single-leg stance	Tandem stance
**Ankle compression socks vs. Control**	0.397	0.445	0.416
**Calf compression socks vs. Control**	0.596	0.221	0.341
**Ankle sleeves vs. Control**	0.503	0.438	0.833

No difference was found between the control socks with shoes and without shoe conditions for all the dynamic and static balance test measurements except right MG following virtual perturbation at direction 1. Larger integrated EMG in the right MG was observed in with shoe condition compared to without shoe conditions (p=0.017, Table. 7). Statistical results of the dynamic balance test measurements in control socks conditions are presented in Table 7 and the statistical results of the static balance test in control socks conditions are presented in Table 8.

**Table 7:** Statistical outcomes of integrated EMG and recovery time during the dynamic balance test in control socks conditions. *P-*values of paired t-test between control socks with shoes VS without shoe conditions. TA- tibialis anterior; PL-peroneal logus; MG- medial gastrocnemius

	Target directions of dynamic balance test							
	1	2	3	4	5	6	7	8
Right TA	0.196	0.426	0.381	0.775	0.803	0.363	0.824	0.492
Right PL	0.202	0.969	0.583	0.542	0.861	0.192	0.545	0.183
Right MG	0.017	0.186	0.298	0.574	0.134	0.525	0.278	0.440
Left TA	0.292	0.817	0.478	0.291	0.959	0.396	0.738	0.132
Left PL	0.313	0.368	0.794	0.272	0.915	0.424	0.504	0.537
Left MG	0.249	0.136	0.118	0.245	0.291	0.869	0.175	0.617
Recovery time	0.288	0.977	0.390	0.958	0.862	0.140	0.469	0.366

**Table 8:** Statistical outcomes of the area of 95% confidence ellipse during static balance test in control socks conditions. *P*-values of paired t-test between control socks with shoes VS without shoe conditions

	Double-leg stance	Single-leg stance	Tandem stance
**Area of 95% confidence ellipse**	0.174	0.113	0.680

### Walking

**Fig. 4:**
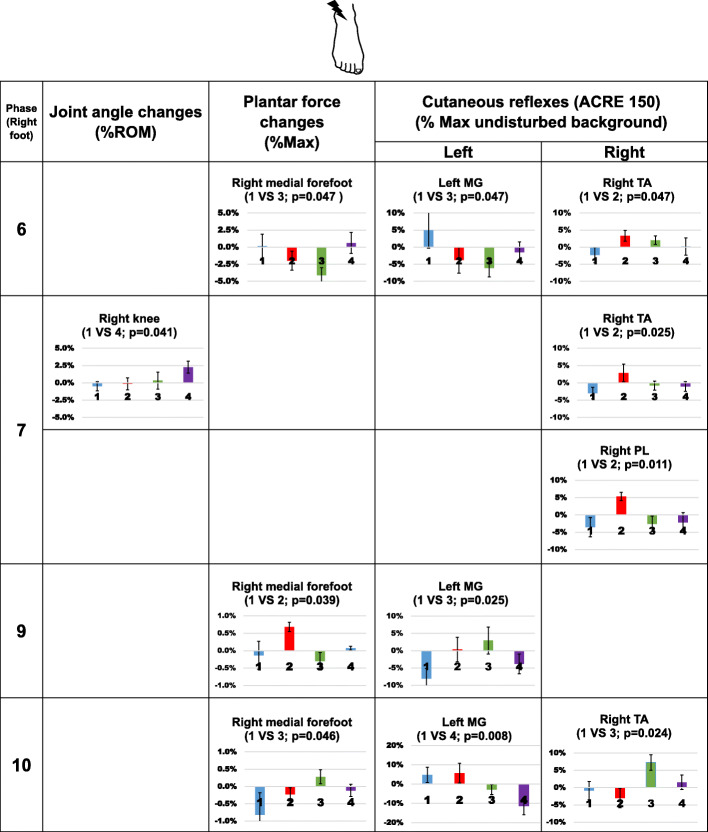
Significant changes in joint angle, plantar force, and net cutaneous reflexes following stimulation to the right ankle crease. In each bar graph, the x-axis represents compression garment conditions, 1(blue): control socks; 2 (red): ankle compression socks; 3 (green): calf compression socks; 4 (purple) ankle sleeves. The condition that significantly differed from the control is indicated at the top of each graph. In the knee angle plot, positive numbers represent extension movement and negative numbers represent flexion movement. Error bars indicate standard error of the group

**Fig. 5:**
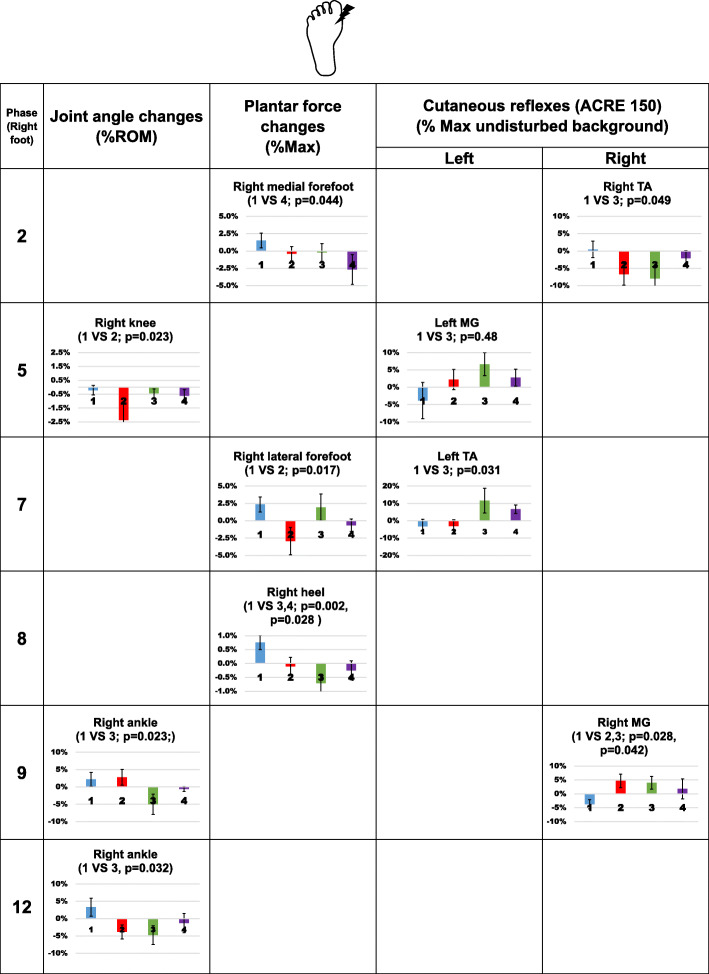
Significant changes in joint angle, plantar force, and net cutaneous reflexes following stimulation to the bottom of the right 1st metatarsal. In each bar graph, the x-axis represents compression garment conditions, 1 (blue): control socks; 2 (red): ankle compression socks; 3 (green): calf compression socks; 4 (purple) ankle sleeves. The condition that significantly differed from the control condition is indicated at the top of each graph. In the knee angle plot, positive numbers represent extension movement and negative numbers represent flexion movement. In the ankle angle plots, positive numbers represent plantar flexion movement and negative numbers represent dorsiflexion movement. Error bars indicate standard error of the group

**Fig. 6:**
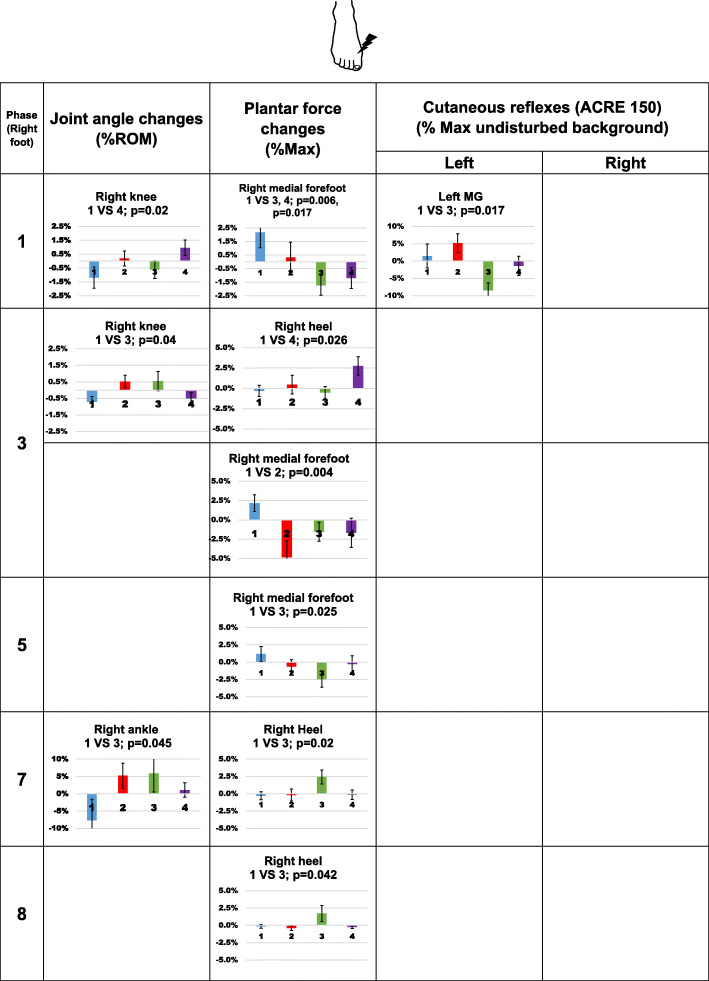
Significant changes in joint angle, plantar force, and net cutaneous reflexes following stimulation to the top of the right 1st metatarsal. In each bar graph, the x-axis represents compression garment conditions, 1 (blue): control socks; 2 (red): ankle compression socks; 3 (green): calf compression socks; 4 (purple) ankle sleeves. The condition that significantly differed from the control condition is indicated at the top of each graph. In the knee angle plots, positive numbers present extension movement and negative numbers represent flexion movement. In the ankle angle plot, positive numbers represent plantar flexion movement and negative numbers represent dorsiflexion movement. Error bars indicate standard error of the group

Joint angle, plantar force and net cutaneous reflexes from each compression condition were all compared to the control condition at 12 phases. Since changes in the joint angle and plantar force directly reflect the changes in gait pattern, we first present significant changes in those measurements followed by cutaneous reflex results, if there is any significant change. Overall results following stimulation from the three different skin locations are shown in Figs. 4, 5 and 6.

#### Stimulation applied to the top of the ankle crease

When stimulation was applied to the top of the ankle crease, altered plantar force was seen under the medial forefoot. At late stance phase (Fig. 4, phase 6), calf compression socks reduced the force by 4.3% of the maximum (*p* = 0.047, d = 0.892). At mid-swing (Fig. 4, phase 9), ankle compression socks increased the force by 0.8% of the maximum (*p* = 0.039, d = 0.789). At late swing, calf compression socks increased the force by 1.1% of the maxima (*p* = 0.046, d = 0.701, Fig. 4, phase 10).

Altered joint angular position was only observed at toe-off in the ankle sleeve condition (Fig. 4, phase 7), knee extension angle increased by 2.8% of the ROM (*p* = 0.041, d = 1.034).

Altered cutaneous net reflex amplitudes were found bilaterally with opposite signs of net reflex in compression garment conditions. During stance to swing transition (Fig. 4, phase 6 and 7), inhibitory net reflexes were changed to facilitatory in the right TA (*p* = 0.047, d = 0.850; *p* = 0.025, d = 0.790) and right PL (*p* = 0.011, d = 1.196) muscles while participants wore ankle compression socks. Calf compression socks altered the signs of net cutaneous reflexes in the left MG muscle at late stance (*p =* 0.047, d = 0.769, Fig. 4, phase 6) and left MG and TA at mid-swing (*p =* 0.025, d = 0.914, phase 9; *p* = 0.024, d = 0.959, phase 10). In the ankle sleeve condition, facilitatory net cutaneous reflex in the left MG muscle changed to an inhibitory response at mid-swing (*p* = 0.008, d = 6.068, Fig. 4, phase 10).

#### Stimulation applied to the bottom of the 1st metatarsal

When stimulation was applied to the bottom foot during mid-stance while wearing ankle compression socks, there was a reduced knee extention angle of 2.2% of the ROM (*p =* 0.024, d = 0.782). When stimulation was applied at swing phase, calf compression socks reduced plantarflexion angle by 7.2% and 8.0% of the ROM at phase 9 and phase 12 (*p* = 0.023, d = 0.877; *p* = 0.032, d = 0.929; Fig. 5).

At early stance (Fig. 5, phase 2), ankle sleeves reduced the force under the right medial forefoot by 4.3% of the maximum (*p* = 0.044, d = 0.773). At stance-swing transition, ankle compression socks decreased force under the right lateral forefoot by 5.4% of the maximum (*p* = 0.017, d = 0.994, Fig. 5, phase 7). Calf compression socks and ankle sleeve decrease the force under the heel by 1.5% (*p* = 0.002, d = 1.462, Fig. 5, phase 8) and 1.1% (*p* = 0.028, d = 0.961, Fig. 5, phase 8) of the maximum respectively.

Altered sign of cutaneous reflexes was observed in the right TA muscle at early stance (*p* = 0.049, d = 0.780, Fig. 5, phase 2), left MG muscle at late stance (*p* = 0.048, d = 0.690, Fig. 5, phase 5), left TA muscle at swing-stance transition (*p* = 0.031, d = 0.733, Fig. 5, phase 7) and right MG muscle (*p* = 0.042, d = 1.110) at phase 9 when participants wearing calf compression socks. Net reflex changes induced by ankle compression socks were found in the right MG muscle at phase 9 (*p* = 0.028, d = 1.156, Fig. 5, phase 9).

#### Stimulation applied to the top of the 1st metatarsal

Following stimulation on the top of the foot at early and mid stance phase, ankle sleeve increased knee extension angle by 2.2% of the ROM (*p =* 0.02, d = 0.662, Fig. 6, phase 1) and calf-compression socks increased knee extension angle by 1.5% of the ROM (*p =* 0.04, d = 0.761, Fig. 6, phase 3).

Force under the right medial forefoot was reduced at heel contact (Fig. 6, phase 1) in calf compression socks and ankle sleeve conditions by 3.8% (*p* = 0.006, d = 1.246) and 3.3% (*p* = 0.017, d = 1.071) of the maximum respectively. Decreased force under medial forefoot was also observed at stance phases in ankle compression socks condition (phase 3, *p* = 0.004, d = 1.265, changed 7% of the maximum) and in calf compression condition (phase 5, *p* = 0.025, d = 0.994, changed 3.6% of the maximum). At phase 3, ankle sleeve increased the force under the heel by 3.2% of the maximum (*p* = 0.026, d = 0.959). When stimulation was applied during toe-off and early swing (Fig. 6, phase 7 and 8), calf compression socks increased the force under the right heel by 2.7% (*p =* 0.02, d = 0.963) and 1.9% (*p* = 0.042, d = 1.462) of the maximum.

Altered sign of net cutaneous reflexes was only observed in the left MG in the calf compression socks condition at heel contact (*p =* 0.017, d = 0.990, Fig. 6, phase 1).

## Discussion

The use of compression socks altered lower limb spinal cord excitability, reflex control, and motor output during walking and balance perturbations. These results extend those obtained in the upper limb and serve to provide a background for continued research into leverage enhanced sensory pathways to amplify human motor behavior.

### Balance

Our results show enhanced performance in dynamic balance tasks but not in static balance tests. With wearing ankle and calf compression socks, recovery time from virtual perturbation at posterior-rightward and posterior-leftward direction were shortened by at least 0.2 s. Reduced integrated EMG in left TA and right PL muscle, was also observed when responding to the perturbation at these two directions.

A few previous studies suggest compression garments can enhance proprioception around the joint and improve movement accuracy in visuomotor [8] or reaching tasks [9]. In Karlsson and Andreasson’s study [18], peroneus longus (plantarlfexor and ankle evertor) muscle response to a simulated ankle tilt was 11 ms faster after applying athletic tape around the ankle of participants with unilateral ankle instability. Athletic tape, commonly used in sports probably provides similar sensory feedback as do compression socks. Both are passive devices that generate altered sensory feedback due to the physical motion of the user. Here, dynamic balance task requires participants to shift their body weight and adjust their center of pressure to follow the target. Reduced total recovery time suggests the ankle- and calf-compression socks can facilitate a more efficient and accurate muscle response to redistribute body weight and recovery from self-induced perturbation, like those found in sports.

In this study, we found TA and PL muscle are more responsive to the changes in condition with altered integrated EMG show at several target directions in all the conditions (Table 5). In a recent study [21], participants performed the same dynamic balance tests under different body weight support conditions. Peak muscle activity of the right TA and left PL muscle occurred at different latencies when perturbation was presented at different directions suggesting these two muscles are likely the prime mover of this self-initiated postural adjustment task [21]. Henry et al. [24] used a translational platform to generate balance perturbation, they observed diagonal modulation pattern in TA and PL with maximal left TA activation occurred after anterior-rightward perturbation and maximal left PL muscle activation occurred after posterior-rightward perturbation. In the current study, reduced recovery time and integrated EMG of left TA and right PL muscle were both observed when perturbation was presented at posterior-rightward and posterior-leftward direction in calf compression condition. Compared to the other two compression garments, calf compression socks likely have stronger effects on enhancing the diagonal modulation pattern in PL and TA muscle and improve the performance in self-initiated posture adjustment tasks.

There was no significant effect of compression garments on double-leg, single-leg and tandem stance performance. Few studies have investigated the effects of compression garments on static balance performance. Michael and colleagues [16] found significantly reduced postural sway during single leg stance (on the dominant foot) with eyes closed and wearing well-fitted compression legging. Michael et al. suggested that compression garments may improve joint positional sense to accommodate the lack of visual feedback. Different from the protocol used by Michael et al., here, participants performed single leg stance test using their non-dominant foot and the compression garments did not cover the entire lower body. Considering these differences, our results may indicate that enhanced sensory feedback from compression garments in the non-dominant foot may not be enough to compensate for the removed visual feedback.

### Walking

We investigated the effect of compression garments on cutaneous reflexes and biomechanical changes in the lower limb during walking. Significant changes in cutaneous reflexes, kinematic and kinetic response amplitudes were observed with the opposite signs in all of the conditions. However, concurrent changes in biomechanical responses and cutaneous reflexes are relatively sparse. Among the three compression garments, bilateral and dynamical changes mostly occurred in the calf compression socks condition.

1. The overall effects of compression on walking

Compared to the control socks condition, cutaneous reflexes and biomechanical responses with the opposite sign were observed with all three types of compression garments during walking. The effects of compression garments on walking biomechanics and metabolic cost were tested by Cheng and Xiong [25]. The authors found that calf compression socks can adjust the knee and ankle kinetics during walking which may be beneficial to lowering the burden of the ankle joint during propulsion. However, there was no effect on muscle activity, gait length, gait frequency and metabolic cost [25]. In the current study, our findings suggest that compression garments can alter interneuronal excitability in the cutaneous pathways as well as change the biomechanical characteristics when perturbation occurred during walking. Reversed cutaneous reflexes have been observed during walking when stimulation was applied to the same nerve at different phases [23, 26] or different skin areas at the same phase [12]. Such phase-dependent and topographical modulation steer the foot away from perturbation.

Not all cutaneous stimulation-induced biomechanical changes could be explained by corresponding changes in muscle activity measured here. For example, stimulation applied to the ankle crease during phase 6 and phase 7, ankle compression socks increased TA muscle activity but not increased dorsiflexion angle (Fig. 4). Similarly, ankle sleeves led to reduced plantar force under medial forefoot when stimulation was applied at the bottom of the foot during phase 2, but no change in cutaneous reflex amplitude was found (Fig. 5). Since we only measured muscle activities in three lower leg muscles on each side, the weak correspondence between biomechanical measurements and cutaneous reflexes may be due to the activities of the other muscles in the lower limb. The effect of overall muscle synergies is also difficult to extract with limited measurement. It is also possible that the walking task in our protocol was not challenging enough to allow or require the expression of exaggerated synergies. Instead of walking on a level surface, walking on an uneven surface requires additional afferent feedback and different neuromuscular modulation [27–29]. Stern and Gottschall [30] compared the gait pattern and muscle activities with different insoles or barefoot during level walking and downhill walking, and found that altered gait patterns due to footwear were more evident at downhill walking condition. Changes in sensation due to compression garments may play a more important role in adjusting neuromuscular strategies during a challenging walking task.

2) Effects of calf compression socks

Among all the results showed in Figs. 4, 5 and 6, some biomechanical changes were observed with altered cutaneous reflex at the same phase, and those changes were all occurred in calf compression sock condition. When stimulation was applied to the ankle crease at the late stance phase (Fig. 4, phase 6), plantar force under the right medial forefoot and muscle response in the left MG muscle (early stance on the left side) were both decreased. Similarly, stimulation applied to the top of the 1st metatarsal at heel contact (Fig. 6, phase 1) reduced MG muscle response on the left side (late stance on the left side) and plantar force under the right medial forefoot. These bilateral responses might delay the push off of the standing leg and weight shifting between legs. Since these changes were found during stance-swing and swing-stance transition of the stimulated leg, the observed changes assist maintaining balance under perturbation by regulating procession to the next phase in the gait cycle.

Compared to the ankle compression socks and ankle sleeves used in this study, the calf compression socks provided graduated pressure around the lower leg. Calf compression socks were as originally used for clinical purposes to improve venous return [31] and prevent deep vein thrombosis [32]. Recently, this has been used in various sports for presumed benefits in performance and post-exercise recovery [3, 33]. Beliard and colleagues [34] suggest that compression garments have positive effects on post-exercise recovery and such effects are not related to how much compression is applied at the ankle or calf. Although the current study is not focused on post-exercise recovery, considering calf compression socks enhanced the sensory feedback from a larger skin area and most of the significant changes were observed in calf compression condition, the area of altered sensory feedback, instead of pressure amplitudes, may play a major role in modulating the gait pattern during walking.

## Conclusions

Compression socks alter sensory feedback transmission to improves performance in corrective balance reactions and amplify spinal cord excitability during walking. That is, during dynamic activity, compression garments enhance sensory feedback that leads to widespread changes in background neural excitability and performance.

Compression socks, especially those around the calf, modulate spinal cord excitability dynamically during walking. The enhancement of sensory feedback seems to generally amplify central reflex gain. The functional relevance of compression garments may be further revealed in more challenging walking (e.g. over uneven terrain) and postural (e.g. in ongoing dynamic perturbation) tasks.

This project, taken together with earlier work on the feet as “sensory antennae” and the role of compression sleeves in the arm, highlights the importance of considering general and specific afferent feedback from the skin in the design of practice and performance apparel.

## Data Availability

The datasets used and/or analyzed during the current study are available from the corresponding author on reasonable request.

## Abbreviations

COP: Center of pressure
mBESS: Modified Balance Error Scoring System
EMG: Electromyography
TA: Tibialis anterior
PL: Peroneus longus
MG: Medial gastrocnemius
FSR: Force sensing resistors
ROM: Range of motion
